# Supplementary material for: Right Ventricular Strain and Left Ventricular Strain Using Speckle Tracking Echocardiography—Independent Prognostic Associations in COPD Alongside NT-proBNP
Source: Diseases. 2025 Oct 16;13(10):344. doi: 10.3390/diseases13100344 (PMC12563488; doi:10.3390/diseases13100344)
Supplement: Supplementary file 1 [file diseases-13-00344-s001.zip › diseases-3857539-supplementary.pdf]

Supplementary Material

Figure S1. Sample size calculation formula

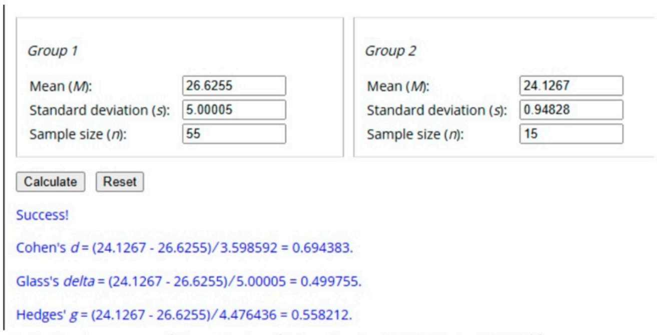

statistical power of the study, Cohen's d= 0.6943=> 69.43%

The parameters used for our calculation were:
Cohen's f effect size =0.7
k (number of group) =4
significance level α =0.05
desired power 1-β =0.80
Based on these parameters, the estimated sample size per control group was approximately n = 15.

2. Additional statistical analysis regarding the present study

Table S1. Baseline demographic, anthropometrics and clinical features in the two groups

Variables	COPD group (n=55)	Control group (n=15)	*p*-Values
Age (years) [a]	68±8.3	65±5.3	0.10
BMI (kg/m^2) [a]	26.6±5	24.1±0.9	0.06
SBP (mmHg) [a]	133±16.8	126±9.1	0.13
DBP (mmHg) [a]	80±9.3	69±8.9	0.06
PP [a]	52.8±13.8	57.2±6.9	0.24
HR (beats/min) [a]	80.6±13.2	78.8±8.5	0.62
Smoking index (pack/year) [a]	27.7±10.3	-	-
Duration of COPD (years) [a]	7±3.5	-	-

[1] *The results were expressed as [a] mean ± SD (standard deviation), [b] absolute frequencies (relative frequencies, %). BMI – body mass index, SBP – systolic blood pressure, DBP – diastolic blood pressure, PP – pulse pressure, HR – heart rate, COPD – chronic obstructive pulmonary disease, *p* – values were obtained by Pearson Correlation, * significant results: $p < 0.05$.

Table S2. Comorbidities, spirometry measurements, and medical therapy were administered to COPD patients according to GOLD grades (1-4)

Variables	COPD GOLD 1	COPD GOLD 2	COPD GOLD 3	COPD GOLD 4	Control group	*p*-Values
**Comorbidities**						
Active smokers [b]	1 (2%)	14 (6%)	6 (10%)	5 (9%)	-	0.22
IHD [b]	1 (2%)	9 (16%)	5 (9%)	5 (9%)	-	0.74
Arrhythmias [b]	0	8 (15%)	4 (7%)	3 (6%)	-	0.65
HBP [b]	3(6%)	21 (38%)	8 (15%)	8 (15%)	-	0.76
Obesity [b]	2 (4%)	12 (22%)	7 (12%)	2 (4%)	-	0.38
Dyslipidemia [b]	3(6%)	13 (24%)	7 (12%)	7 (12%)	-	0.65
DM type 2 [b]	1 (2%)	3(6%)	3(6%)	3 (6%)	-	0.50
**Spirometry**						
FEV1 % [a]	85±3.5	65.7±8.5	45.2±3.4	27±7.3	-	0.001*
FVC (L) [a]	4.3±1.2	3.6±0.2	3.1±0.1	2.6±0.1	-	0.001*
**Medication**						
Antiplatelet drugs [b]	1 (2%)	13 (24%)	5 (9%)	4 (7%)	-	0.85
Anticoagulant [b]	0	4 (7%)	3(6%)	3 (6%)	-	0.50
Statins [b]	2 (4%)	4 (7%)	5 (9%)	4 (7%)	-	0.26
RAAS inhibitors [b]	3 (6%)	20 (37%)	7 (12%)	7 (12%)	-	0.37
CCBs [b]	1 (2%)	9 (16%)	5 (9%)	2 (4%)	-	0.21
Diuretics [b]	1 (2%)	18 (15%)	8 (15%)	8 (15%)	-	0.27
BB [b]	2 (4%)	19 (35%)	8 (15%)	8 (15%)	-	0.67
LAMA [b]	1 (2%)	7 (13%)	2 (4%)	4 (7%)	-	0.61
SABA [b]	1 (2%)	9 (16%)	6 (10%)	7 (13 %)	-	0.17
LABA/ICS [b]	1 (2%)	3 (5 %)	6 (10%)	3 (5 %)	-	0.91
LAMA/LABA [b]	1 (2%)	17 (30%)	6 (10%)	1 (2%)	-	0.039*
LAMA/LABA/ICS [b]	0	0	3 (5%)	4 (7%)	-	0.006*
Mucolytic agents [b]	2 (4%)	20 (37%)	12 (22%)	7 (12%)	-	0.48

[1] The results were expressed as [a] mean ± SD (standard deviation), [b] absolute frequencies (relative frequencies, %). IHD – ischemic heart disease, HBP – High blood pressure, DM – diabetes mellitus, FEV1 - forced expiratory volume in one second, FVC - forced vital capacity, RAAS – renin-angiotensin-aldosterone system, CCBs – calcium channel blockers, BB – beta blockers, LAMA – long-acting muscarinic agonist, SABA – short-acting ß2 agonist, LABA – long-acting ß2 agonist, ICS – inhaled corticosteroids; * significant results: *p* – values were obtained by Pearson Correlation, * $p < 0.05$.

Table S3 shows the statistical analysis of the laboratory parameters observed between the control group and patients diagnosed with COPD.

Table S3. Laboratory parameter values in the control group and the COPD group

Variables	COPD group (n=55)	Control group (n=15)	*p*-Values
Hb (g/dl)	14.3±1.9	13.9±1.0	0.51
Ht (%)	44.1±5.7	43.7±4.2	0.80
Tr (x1000/mm^3)	228.6±74.0	250.7±88.7	0.33
Ne (%)	65.9±13.5	61.3±10.5	0.22
NLR (%)	4.46±3.1	2.62±0.5	0.030*
Eo (cells/μL)	322±200	130±57.6	0.001*
CRP (mg/L)	22.1±24.9	3±0.7	0.004*
Fasting glucose (mg/dl)	108.3±20.6	90.8±10.1	0.002*
Uric acid (md/dl)	5.9±1.6	5.2±0.9	0.11
LDLc (mg/dl)	90.2±33.4	90.8±14.1	0.94
MDA (nmol/ml)	2952.4±948.3	1442.0±959.5	0.001*
GSH (nmol/ml)	6.9±2.0	8.6±2.4	0.008*
GSSG (nmol/ml)	1.2±0.50	0.7±0.3	0.004*
GSH/GSSG	5.9±2.7	14.6±8.65	0.001*
VEGF (pg/ml)	242.1±155.3	159.8±29.2	0.04
IL-6 (pg/ml)	156.9±137.1	93.1±42.7	0.08
Casp 3 (pg/ml)	240.8±119.2	170.1±75.0	0.03*
Casp 9 (pg/ml)	200.7±64.7	172.4±64.7	0.13
NT-proBNP (pg/ml)	480.0±471.6	78.3±17.9	0.002*

[1] The results were expressed as mean ± SD (standard deviation). Hb - hemoglobin, Ht - hematocrit, Tr - thrombocytes, Ne - neutrophils, NLR - neutrophil/lymphocyte ratio, Eo - eosinophils, CRP - C-reactive protein, LDLc - low-density lipoprotein cholesterol, MDA - malondialdehyde, GSH - reduced glutathione, GSSG - oxidized glutathione, GSH/GSSG - reduced glutathione/oxidized glutathione ratio, IL-6 – interleukin-6, Casp 3 – Caspase-3, Casp 9 – Caspase-9; * significant results; *p* – values were obtained by Pearson Correlation, * $p < 0.05$.

Table S2 shows statistically significant differences in laboratory parameters according to COPD severity, based on GOLD grades and severity of airflow obstruction in COPD.

Table S4. Laboratory parameter values based on GOLD grades (1-4)

Variables	COPD GOLD 1	COPD GOLD 2	COPD GOLD 3	COPD GOLD 4	*p*-Values
Hb (g/dl)	14.6±1.1	14.3±1.6	14.6±2.8	13.7±1.9	0.74
Ht (%)	46.1±1.9	44.5±4.5	43.6±8.4	43.1±5.7	0.81
Tr (x1000/mm^3)	239.5±50.2	238.9±80.3	213.9±79	214.8±58.5	0.69
Ne (%)	52.±12.6	66.4±13.3	64.8±12.7	71.5±12.9	0.1
NLR (%)	2.1±2	4.1±3	4.3±2.8	6.6±3.4	0.05*
Eo (cells/μL)	361.5±139.9	293.3±208.4	338.8±207.1	365±201.7	0.74
CRP (mg/L)	13.7±20	21.1±16	26.3±24.9	45.2±27.2	0.005*

Fasting glucose (mg/dl)	122.6±51.6	103.8±10.1	112.6±14.1	110.3±28.1	0.25
Uric acid (md/dl)	5.9±1.6	5.5±1.9	6.9±1.6	5.7±2.3	0.06
LDLc (mg/dl)	103.2±25.4	89.7±30.6	103.5±43	69.4±21	0.08
MDA (nmol/ml)	2606.4±996.3	2751.7±760.6	2840.3±961.3	3125.7±1007.4	0.56
GSH (nmol/ml)	7.1±1.4	6.6±1.9	7±2.0	4.9±2.5	0.12
GSSG (nmol/ml)	0.7±0.3	0.6±0.2	0.6±0.5	0.6±0.5	0.80
GSH/GSSG	4±2.3	5.9±2.9	6±2.8	6.2±2.2	0.56
VEGF (pg/ml)	257±111.8	234±121.3	301±138.1	282.3±133.9	0.33
IL-6 (pg/ml)	160±218.6	137.1±120.1	152.7±138	217.8±148	0.47
Casp 3 (pg/ml)	196.2±37.2	243.5±128.8	260±144.2	225.3±74.3	0.78
Casp 9 (pg/ml)	183.8±32.1	196.8±59.1	208.4±144.2	208.5±79.3	0.89
NT-proBNP (pg/ml)	148.5±39.5	301.2±111.3	598.6±685.8	958.9±496.2	<0.001*

[1] Results were expressed as mean ± SD (standard deviation). Hb - hemoglobin, Ht - hematocrit, Tr - thrombocytes, Ne - neutrophils, NLR - neutrophil/lymphocyte ratio, Eo - eosinophils, CRP - C- reactive protein, LDLc - low-density lipoprotein cholesterol, MDA - malondialdehyde, GSH - reduced glutathione, GSSG - oxidized glutathione, GSH/GSSG - reduced glutathione/oxidized glutathione ratio, IL-6 – interleukin-6, Casp 3 – Caspase-3, Casp 9 – Caspase-9; * significant results; *p* – values were obtained by Pearson Correlation, * $p < 0.05$.

Table S5. Matrix of Spearman's rank correlations between MDA, GSH, GSSG, GSH/GSSG, VEGF, IL-6, Caspase-3, Caspase-9, NT-proBNP, and clinical characteristics, laboratory parameters, spirometry, and echocardiographic parameters

**Variables**	**MDA**	**GSH**	**GSSG**	**GSH/GSSG**	**Casp 3**	**Casp 9**	**NT-proBNP**
Age, years	0.05; 0.70	**-0.27; 0.05***	0.2; 0.13	**-0.37; 0.005***	**0.32; 0.01***	0.01; 0.94	0.17; 0.19
Smoking index (pack/year)	0.04; 0.71	-0.07; 0.56	0.16; 0.21	**-0.27; 0.03***	0.15; 0.26	0.25; 0.06	0.23; 0.51
Duration of COPD (years)	0.02; 0.86	-0.24; 0.75	0.05; 0.68	0.18; 0.18	0.21; 0.11	0.05; 0.71	**0.56; 0.001***
NLR (%)	-0.01; 0.94	-0.07; 0.58	-0.05; 0.69	0.22; 0.09	0.13; 0.34	0.02; 0.84	**0.29; 0.03***
CRP (mg/L)	-0.04; 0.76	-0.08; 0.53	-0.18; 0.18	0.23; 0.07	0.58; 0.67	-0.14; 0.29	**0.38; 0.001***
pH	0.01; 0.96	-0.11; 0.39	-0.01; 0.89	-0.07; 0.57	-0.14; 0.28	-0.05; 0.71	**-0.64; 0.001***
PCO_2 (mmHg)	-0.19; 0.15	0.23; 0.08	0.01; 0.96	0.27; 0.06	0.15; 0.27	0.11; 0.40	**0.74; 0.001***
HCO_{3^-} (mEq/L)	-0.12; 0.12	0.05; 0.35	-0.10; 0.67	0.17; 0.44	0.02; 0.85	0.04; 0.76	**0.59; 0.001***
PaO_2 (mmHg)	0.10; 0.46	-0.03; 0.77	-0.13; 0.33	0.03; 0.80	-0.12; 0.37	-0.18; 0.17	**-0.64; 0.001***
Sat O_2	-0.07; 0.60	0.03; 0.79	-0.13; 0.32	-0.04; 0.77	-0.18; 0.17	-0.23; 0.37	**-0.56; 0.001***
6MWT (m)	0.11; 0.42	0.22; 0.09	-0.03; 0.82	-0.23; 0.07	-0.15; 0.26	-0.05; 0.68	**-0.57; 0.001***
FEV1 (%)	0.13; 0.32	-0.08; 0.55	-0.04; 0.74	-0.10; 0.42	-0.08; 0.54	-0.01; 0.97	**-0.68; 0.001***
FVC (L)	0.10; 0.45	**0.27; 0.05***	0.08; 0.53	**0.27; 0.04***	-0.02; 0.84	-0.04; 0.72	**-0.65; 0.001***
GLS VS (%)	0.01; 0.98	0.11; 0.39	-0.06; 0.63	0.24; 0.06	0.13; 0.32	-0.05; 0.71	-0.54; 0.001*
S' RV (cm/sec)	0.08; 0.55	-0.02; 0.86	0.01; 0.91	-0.08; 0.55	-0.08; 0.54	-0.01; 0.44	**-0.47; 0.001***

TAPSE (mm)	0.17; 0.25	-0.15; 0.24	-0.10; 0.44	-0.07; 0.59	-0.05; 0.71	-0.04; 0.73	**-0.53**; **0.001***
FAC-RV (%)	0.01; 0.93	-0.01; 0.92	-0.02; 0.83	-0.13; 0.34	-0.21; 0.11	-0.11; 0.41	**-0.75**; **0.001***
RVFWSL %	-0.01; 0.92	0.19; 0.15	0.01; 0.90	0.22; 0.09	0.15; 0.27	0.07; 0.58	**-0.66**; **0.001***
RV4CSL %	-0.04; 0.750	0.22; 0.09	0.01; 0.97	0.26; 0.05	0.13; 0.3	0.09; 0.48	**-0.66**; **0.001***
RA aria (cm^2)	0.04; 0.76	0.27; 0.06	0.12; 0.35	0.02; 0.83	-0.01; 0.90	-0.04; 0.75	0.12; 0.37
RA volume (mL/m^2)	0.13; 0.31	0.10; 0.44	0.07; 0.59	-0.01; 0.96	0.10; 0.44	0.21; 0.11	0.13; 0.34
LA volume (mL/m^2)	-0.02; 0.87	0.12; 0.37	0.01; 0.90	-0.02; 0.87	0.09; 0.49	-.0.01; 0.98	0.09; 0.50
LASr (%)	0.05; 0.71	0.01; 0.92	0.01; 0.91	0.13; 0.32	-0.03; 0.81	0.03; 0.79	-0.05; 0.66
LAScd (%)	0.08; 0.53	-0.06; 0.62	-0.07; 0.60	-0.03; 0.80	0.13; 0.32	0.14; 0.28	0.06; 0.65
LASct (%)	0.04; 0.74	-0.10; 0.45	0.06; 0.63	-0.08; 0.55	0.05; 0.41	-0.12; 0.45	0.03; 0.77
PASP (mmHg)	0.04; 0.73	-0.03; 0.82	-0.19; 0.15	0.16; 0.24	0.02; 0.84	0.08; 0.54	0.65; 0.001*

[1] The results were expressed as mean ± SD (standard deviation). ABG – arterial blood gas, MDA - malondialdehyde, GSH - reduced glutathione, GSSG - oxidized glutathione, GSH/GSSG - reduced glutathione/oxidized glutathione ratio, IL-6 – interleukin-6, Casp 3 – Caspase-3, Casp 9 – Caspase-9, NLR - neutrophil/lymphocyte ratio, CRP - C-reactive protein, PCO2 - partial pressure of carbon dioxide, PaO2 - partial pressure of oxygen, HCO3 - bicarbonate, SatO2 - oxygen saturation, 6MWT- 6 minute walking test, FEV1 - forced expiratory volume in the first second, FVC - forced vital capacity, FEV1/FVC - forced expiratory volume in the first second (FEV1) relative to forced vital capacity (FVC), LVEF - left ventricular ejection fraction, GLS LV avg - mean global longitudinal strain of the LV, RV - right ventricle, S' RV - systolic longitudinal contraction of the RV, TAPSE - systolic motion of the tricuspid annulus plane, LW - lateral wall, FAC – fractional aria change, RVFWSL - RV free wall longitudinal strain , RV4CSL – RV four chamber longitudinal strain, RA – right atrium, LA – left atrium, LASr – reservoir function of the LA, LAScd – conduit function of the LA, LASct – contraction function of the LA. * significant results; *p* – values were obtained by Pearson Correlation, * $p < 0.05$.

3. Oxidative stress markers

Glutathione is usually found in its reduced form in the body (GSH). GSH protects against lung peroxidation and is critical for inhibiting lung destruction in COPD. GSH is further converted to oxidized glutathione (GSSG) under oxidative stress via glutathione peroxidase. GSSG can be reduced to GSH through glutathione reductase, which maintains a redox status and an intracellularly increased GSH/GSSG ratio. The GSH/GSSG ratio is also used to indicate oxidative stress [34]. Clinical trials published to date showed decreased antioxidants (GSH) levels and reduced redox status (GSH/GSSG) levels in COPD patients when comparing healthy participants to those diagnosed with COPD [1]. In our study, the serum GSH levels were lower in patients diagnosed with COPD compared to the healthy control population. No significant differences in GSH were observed in the different GOLD severity grades of COPD in this study or in the literature [1,2].

However, the GSSG value was higher in patients diagnosed with COPD compared to the healthy population in the control group, resulting in a lower GSH/GSSG ratio in patients diagnosed with COPD. This points to a high level of oxidative stress in this group of patients. No statistically significant differences were observed between exacerbation frequency and serum GSH, GSSG, or GSH/GSSG redox status levels in this study. In addition, GSH, GSSG, or GSH/GSSG redox status levels were not able to distinguish severe/very severe stages of COPD from mild/moderate stages of COPD. One limitation was most likely

the reduced number of patients. However, data in the literature show that GSH and GSH/GSSG ratios decreased proportionally to the frequency of COPD exacerbations [1]. Spearman's rank correlation coefficient showed a significant negative correlation between lower serum GSH levels and lower GSH/GSSG ratio, and older ages in patients diagnosed with COPD. It is confirmed that antioxidant defense systems become more deficient with advanced age, a fact also supported by the latest review published in this regard [3]. Moreover, the correlation coefficient shows a significant negative relationship between low serum GSH/GSSG ratio values and smoking index in this patient category. Once again, these results confirmed the negative impact of smoking on antioxidant defense mechanisms and its direct involvement in ROS generation [4].

MDA is an essential marker used to describe systemic lipid peroxidation, a key indicator of oxidative stress, and plays a key role in COPD pathogenesis. Lipid peroxidation via enzymatic or non-enzymatic mechanisms is directly involved in pulmonary oxidative tissue damage. Numerous studies have reported increased MDA levels in the smoking population, as well as in patients with COPD [2,5,6]. In the present study, MDA levels were significantly higher in patients diagnosed with COPD compared to the control group, similar results having been described in the literature [6–8]. Reduced levels of antioxidants and increased metabolite levels derived from lipid peroxidation are associated with reduced lung function in the general population [2]. Thus, patients with COPD have a much higher oxidative stress value than healthy patients. In a recently published study, Promsrisuk described a significant increase in MDA level directly proportional to COPD severity and pointed out an inversely proportional correlation between plasma MDA level and lung function determined by spirometry [9]. This study detected no significant differences in MDA compared with the GOLD severity grades (1-4) in patients diagnosed with COPD or in the frequency of exacerbations per year. Additionally, MDA was not able to distinguish severe/very severe stages of COPD from mild/moderate stages. A future study with a large population is required for better clarification.

Cysteine proteases, such as caspases, are essential in regulating lung cell apoptosis, and several mechanisms are involved [10]. The first pathway is activated by an extracellular signal. Pro-Caspase-8 is activated into Caspase-8, which in turn activates Caspase-3 (Casp 3), and ultimately results in bronchial epithelial cell apoptosis. The second, intrinsic, mitochondrial pathway in response to increased levels of chemical stressors is the release of Cytochrome C from mitochondria. Cytochrome C activates Caspase-9, which, in turn, activates Caspase-3 and thus initiates apoptosis in the bronchial epithelial cells. Apoptosis is an essential mechanism for maintaining normal tissue homeostasis, and any imbalance that influences the apoptosis-proliferation balance in lung tissue may contribute to COPD pathogenesis [10,11]. There is a chronic loss of both epithelial and endothelial lung cells due to altered programmed apoptosis. The presence of activated Caspase-3 showed increased values mainly in the lung parenchyma [12,13]. This study highlighted an increased serum level of Caspase-3 in COPD patients compared to the control group. Nevertheless, there were no significant differences in Caspase-3 values according to the various GOLD grades and severity of airflow obstruction, and Caspase-3 was not able to distinguish severe/very severe stages of COPD from mild/moderate stages. A future study with a large population is needed for better clarification. However, patients with frequent COPD exacerbations per year had significantly higher Caspase-3 values than patients with one exacerbation. Using a multiple linear regression model, a significant relationship has been demonstrated between the increase in the frequency of exacerbations and serum Caspase-3 levels. Using Spearman's rank coefficient, Caspase-3 had a significant correlation with the COPD patients' age. Increased proteolytic and apoptotic activity in COPD patients is influenced by lung pathology per se and declining antiproteolytic defense mechanisms with increasing age.

References

1. Taniguchi, A.; Tsuge, M.; Miyahara, N.; Tsukahara, H. Reactive Oxygen Species and Antioxidative Defense in Chronic Obstructive Pulmonary Disease. *Antioxidants (Basel)* **2021**, *10*, 1537, doi:10.3390/antiox10101537.
2. Singh, S.; Verma, S.K.; Kumar, S.; Ahmad, M.K.; Nischal, A.; Singh, S.K.; Dixit, R.K. Evaluation of Oxidative Stress and Antioxidant Status in Chronic Obstructive Pulmonary Disease. *Scand J Immunol* **2017**, *85*, 130–137, doi:10.1111/sji.12498.
3. Detcheverry, F.; Senthil, S.; Narayanan, S.; Badhwar, A. Changes in Levels of the Antioxidant Glutathione in Brain and Blood across the Age Span of Healthy Adults: A Systematic Review. *NeuroImage: Clinical* **2023**, *40*, 103503, doi:10.1016/j.nicl.2023.103503.
4. Cha, S.-R.; Jang, J.; Park, S.-M.; Ryu, S.M.; Cho, S.-J.; Yang, S.-R. Cigarette Smoke-Induced Respiratory Response: Insights into Cellular Processes and Biomarkers. *Antioxidants* **2023**, *12*, 1210, doi:10.3390/antiox12061210.
5. Bartoli, M.L.; Novelli, F.; Costa, F.; Malagrinò, L.; Melosini, L.; Bacci, E.; Cianchetti, S.; Dente, F.L.; Di Franco, A.; Vagaggini, B.; et al. Malondialdehyde in Exhaled Breath Condensate as a Marker of Oxidative Stress in Different Pulmonary Diseases. *Mediators of Inflammation* **2011**, *2011*, 1–7, doi:10.1155/2011/891752.
6. Ingale P, Rai P, Salunkhe V, Awad NT. Is Malondialdehyde(MDA) Used as a Oxidative Stress Marker in Chronic Obstructive Pulmonary Disease(COPD) & Cigarette Smokers. *JK Science* **2022**, *24(4)*.
7. Moussa, S.B.; Sfaxi, I.; Tabka, Z.; Saad, H.B.; Rouatbi, S. Oxidative Stress and Lung Function Profiles of Male Smokers Free from COPD Compared to Those with COPD: A Case-Control Study. *Libyan Journal of Medicine* **2014**, *9*, 23873, doi:10.3402/ljm.v9.23873.
8. Saeed, Z.H.; El Hakim, M.A.E.A.; Mohamed, N.R. Chronic Obstructive Pulmonary Disease in Non-Smokers: Role of Oxidative Stress. *Egypt J Bronchol* **2021**, *15*, 40, doi:10.1186/s43168-021-00088-5.
9. Promsrisuk, T.; Boonla, O.; Kongsui, R.; Sriraksa, N.; Thongrong, S.; Srithawong, A. Oxidative Stress Associated with Impaired Autonomic Control and Severity of Lung Function in Chronic Obstructive Pulmonary Disease Patients. *J Exerc Rehabil* **2023**, *19*, 75–84, doi:10.12965/jer.2244586.293.
10. Demedts, I.K.; Demoor, T.; Bracke, K.R.; Joos, G.F.; Brusselle, G.G. Role of Apoptosis in the Pathogenesis of COPD and Pulmonary Emphysema. *Respir Res* **2006**, *7*, 53, doi:10.1186/1465-9921-7-53.
11. Dey, T.; Kalita, J.; Weldon, S.; Taggart, C.C. Proteases and Their Inhibitors in Chronic Obstructive Pulmonary Disease. *JCM* **2018**, *7*, 244, doi:10.3390/jcm7090244.
12. Imai, K.; Mercer, B.A.; Schulman, L.L.; Sonett, J.R.; D'Armiento, J.M. Correlation of Lung Surface Area to Apoptosis and Proliferation in Human Emphysema. *Eur Respir J* **2005**, *25*, 250–258, doi:10.1183/09031936.05.00023704.
13. Pandey, K.C.; De, S.; Mishra, P.K. Role of Proteases in Chronic Obstructive Pulmonary Disease. *Front. Pharmacol.* **2017**, *8*, 512, doi:10.3389/fphar.2017.00512.
